# Integrated Bulk and Single-Cell Transcriptomic Analysis Identifies a Reproducible SASP-Related Three-Gene Panel and Prioritizes CFB as a Fibroblast-Associated Marker in Rheumatoid Arthritis

**DOI:** 10.3390/genes17070736

**Published:** 2026-06-26

**Authors:** Jiang Zhang, Ming Li, Xuancheng Jin, Xiaojing Huang

**Affiliations:** 1 Shenzhen University Health Science Center, Shenzhen University, Shenzhen 518060, China; 2 Department of Rehabilitation Medicine, Shenzhen University General Hospital, Shenzhen 518055, China

**Keywords:** rheumatoid arthritis, senescence-associated secretory phenotype, single-cell analysis, transcriptomics, bioinformatics, *CFB*, fibroblasts

## Abstract

Background/Objectives: Rheumatoid arthritis (RA) is a chronic inflammatory autoimmune disease in which senescence-associated secretory phenotype (SASP)-related transcriptional programs may contribute to synovial inflammation and fibroblast activation. This study aimed to integrate bulk transcriptomic cohorts with the gene-expression component of a single-nucleus multimodal dataset to identify reproducible SASP-related candidate biomarkers and prioritize fibroblast-associated signals in RA. Methods: SASP-score correlation analysis, differential expression analysis, cross-cohort evaluation, logistic regression, gene set enrichment analysis, single-nucleus transcriptomic characterization, predicted regulatory network analysis, and RT-qPCR assessment were performed. GSE89408 was used as the discovery bulk transcriptomic cohort, GSE77298 as the external evaluation cohort, and GSE243917 as the single-nucleus gene-expression dataset. Results: Fibroblasts showed relatively high SASP-related transcriptional scores within the RA single-nucleus dataset. The cross-cohort evaluation retained *RCAN1*, *IRF1*, *CFB*, and *TIMP1* as reproducibly elevated candidates. Among all 15 non-empty panels derived from these four genes, the *RCAN1/IRF1/CFB* panel achieved the highest five-fold cross-validated AUC in GSE89408 and tied for the highest external AUC in GSE77298. Adding *TIMP1* did not improve the external AUC or the 95% confidence interval. Among the retained genes, *CFB* showed the strongest fibroblast-associated expression pattern and SASP-score correlation. The RT-qPCR analysis provided preliminary mRNA-level support for the increased expression of *RCAN1*, *IRF1*, and *CFB* in the RA-related cell samples. Conclusions: *RCAN1*, *IRF1*, and *CFB* form a reproducible SASP-related candidate panel in RA, with *CFB* prioritized as a fibroblast-associated marker. Further protein-level, functional, and controlled single-cell validation studies are required.

## 1. Introduction

Rheumatoid arthritis (RA) is a chronic inflammatory disorder characterized by synovial swelling, cartilage destruction, and progressive joint deformity, with a prevalence of about 1% worldwide [1]. The development and progression of RA involve a complex interplay of genetic, environmental, and immunologic factors [2]. Despite advances in treatment strategies, such as disease-modifying antirheumatic drugs (DMARDs) and related biologics, the heterogeneous nature of RA leads to a suboptimal treatment response in many patients. Therefore, identifying dependable biomarkers remains important for improving disease characterization and prognosis.

In chronic inflammation, tissue remodeling, tissue damage, and age-related disorders, the senescence-associated secretory phenotype (SASP) exerts important effects through the secretion of pro-inflammatory cytokines, chemokines, and growth factors [3]. In RA, senescent synovial fibroblasts can display an increased expression of inflammatory mediators such as IL6, CXCL8, and MMP3 [4]. In parallel, single-cell studies have demonstrated marked heterogeneity among synovial fibroblast subsets in RA, supporting the concept that fibroblasts have distinct pathogenic functions in the inflamed synovium [5]. Therefore, understanding the SASP-related transcriptional features in RA may help clarify aspects of chronic inflammation and joint destruction.

This study integrates single-cell and bulk transcriptomic datasets to identify reproducible SASP-related candidate markers in RA and to prioritize fibroblast-associated signals for further validation.

## 2. Materials and Methods

### 2.1. Data Sources and Preparation

Bulk transcriptomic and single-cell/single-nucleus transcriptomic datasets were collected from the GEO website (https://www.ncbi.nlm.nih.gov/geo/, accessed on 5 March 2025). GSE89408 (Platform: GPL11154) was used as the main bulk transcriptomic cohort and contains 218 synovial samples spanning normal controls (*n* = 28), osteoarthritis (*n* = 22), arthralgia (*n* = 10), undifferentiated arthritis (*n* = 6), early RA (*n* = 57), and established RA (*n* = 95) [6]. For model development, normal samples and RA samples were used to minimize diagnostic ambiguity, while the additional non-RA categories were retained for disease-context analyses. GSE77298 (Platform: GPL570) served as the primary external evaluation cohort and includes 16 RA and 7 healthy control synovial samples [7]. GSE7307 (Platform: GPL570), which consists of 6 RA and 5 healthy control synovial samples, was used as an additional small supportive expression–validation cohort.

The gene-expression component of the single-nucleus multimodal dataset GSE243917 (Platform: GPL24676) contained synovial tissue samples from 11 RA donors and one OA donor [8]. Since only one OA donor was available and no healthy synovial controls were included, the OA donor was not used as a formal comparison group. Accordingly, this dataset was used primarily to characterize relative SASP-associated differences among cell populations and *CFB*-related fibroblast states within RA synovial tissue rather than to infer disease-specific differences between RA and non-RA synoviums. Two publicly available datasets (GSE200815 and GSE216651) generated on the GPL24676 platform were integrated as an independent single-cell validation cohort. GSE200815 comprises RA synovial tissue samples, and GSE216651 includes healthy samples from the infrapatellar fat pad (IPFP) and synovium. This integrated validation cohort was used to corroborate cell-type-level SASP patterns and fibroblast-associated biomarker expression. In addition, GSE243917 and GSE216651 were compared as a supportive exploratory analysis for disease-control differences in the lead biomarker. Since the healthy samples in GSE216651 included IPFP/synovial tissue and came from a different study, this comparison should be interpreted as supportive rather than definitive disease-specific validation.

Additionally, SASPRGs were extracted from the GeneCards library (https://www.genecards.org/, accessed on 24 May 2025) (threshold setting: relevance score > 7) and the published literature [9,10], and 463 SASPRGs were obtained after merging and deduplication.

### 2.2. Single-Cell Data Processing and Cellular Annotation

To extract key information from the gene-expression component of the single-nucleus multimodal dataset, we used the Seurat R package (version 5.1.0) for transcriptomic analysis [11]. Cells/nuclei were retained if they had 200–6000 detected genes, nCount_RNA < 25,000, and mitochondrial gene proportion < 20%. Potential doublets were further removed using DoubletFinder (version 2.0.4) [12]. Data normalization was then performed, after which the 2000 genes with the highest variability were selected for subsequent analyses. Subsequently, the IntegrateData function was used to address batch effects, followed by principal component analysis (PCA) to reduce data dimensionality. Next, the FindNeighbors and FindClusters functions were applied to conduct unsupervised cluster analysis (resolution = 0.5). Cell annotation was conducted by comparing the expression levels of cell-type-specific marker genes obtained from integrating relevant published studies [13] and the CellMarker repository (http://117.50.127.228/CellMarker/, accessed on 24 May 2025) across the identified clusters. The following canonical markers were used for each cell type: B cells (*RALGPS2* and *CD79A*), T cells (*CD2* and *CD3D*), macrophages (*CD14*, *CD163*, *PLAUR*, and *LYZ*), endothelial cells (*VWF* and *CD34*), mural cells (*ACTA2* and *MCAM*), and fibroblasts (*ISLR*, *PDGFRA*, *PDPN*, and *PRG4*). Subsequently, the same analytical workflow was applied to the independent single-cell validation cohorts (GSE200815 and GSE216651).

### 2.3. Exploratory Identification of the Cell Population with Relatively High SASP-Related Scores

Five independent enrichment scoring algorithms were utilized to comprehensively assess SASP activity at the single-cell level: AUCell, UCell, singscore, ssGSEA, and the built-in AddModuleScore function in Seurat. For each algorithm, raw SASP scores were first calculated for every cell. Subsequently, min-max normalization was applied to linearly map the raw scores of each algorithm to a [0, 1] interval using the formula:

normalized_score = (x −min (x)) / (max(x)− min (x))

The arithmetic mean of the five normalized scores was calculated for each cell and used as the integrated SASP score. Based on these integrated scores, the cell population with the highest SASP-related scores within the RA single-cell dataset was selected for downstream exploratory analysis and was further divided into high- and low-score groups based on the average SASP score. The same procedure was performed in GSE200815 to corroborate these findings. To compare the expression profiles between the two groups, differential expression analysis was conducted using FindMarkers, with criteria established at log_2_ fold change (FC) > 0.5 and adj.*p* < 0.05. The clusterProfiler R package (version 4.7.1.001) was applied for Gene Ontology (GO) and Kyoto Encyclopedia of Genes and Genomes (KEGG) analyses to investigate the probable biological roles (*p* < 0.05) of the up-regulated genes between the two groups [14]. The STRING database (http://string.embl.de/, accessed on 28 May 2025) was used to construct a protein–protein interaction (PPI) network for these up-regulated genes to analyze their connections (confidence score ≥ 0.7).

### 2.4. Identification of SASP-Positive-Related Genes

To determine SASP-positive-related genes, the average SASP score for every single cell was computed. Next, Spearman correlation analysis was performed to evaluate the correlation between the cellular mean SASP score and the expression level of each SASPRG. For preliminary screening, genes with a correlation coefficient (Cor) > 0.1 and a raw *p*-value < 0.05 were preliminarily defined as SASP-positive-associated genes. To reduce false positive results caused by multiple testing, the Benjamini–Hochberg (BH) method was applied for false discovery rate (FDR) correction. Genes with an adjusted FDR (q-value) < 0.05 were finally recognized as statistically significant SASP-positive-associated genes.

### 2.5. Differential Expression Analysis

For RA-control DEG detection in GSE89408, the DESeq2 R package (version 1.38.3) was used (log_2_FC > 0.5, adj.*p* < 0.05) [15], and results were plotted as a volcano diagram with ggplot2 (version 3.3.6).

### 2.6. Identification and Cross-Cohort Evaluation of SASP-Related Candidate Biomarkers

Firstly, the intersection among up-regulated genes between high- and low-score groups in fibroblasts, SASP-positive-related genes, and the up-regulated genes between RA and control groups were taken, and the intersection genes were defined as candidate genes. Candidate genes were then evaluated in GSE89408 using 5-fold stratified cross-validation with logistic regression, followed by cross-cohort external evaluation in GSE77298. For cross-platform validation, the corresponding GPL570 probes in GSE77298 were mapped to each candidate gene and expression values were standardized within each dataset using z-scores. Genes showing significantly higher expression in RA than in controls in both GSE89408 and GSE77298, with directionally consistent changes, were considered reproducibly elevated biomarkers. Since GAPDH is a housekeeping gene, its external reproducibility was examined explicitly. All possible panels derived from the four reproducibly elevated genes (*RCAN1*, *IRF1*, *CFB* and *TIMP1*) were subsequently compared, and a three-gene candidate panel focusing on *RCAN1*, *IRF1*, and *CFB* was retained for downstream analyses based on model parsimony, cross-validation performance, external performance and downstream single-cell interpretability. Since GSE77298 was used for cross-cohort panel comparison, the external AUC should be interpreted as independent external evaluation rather than definitive prospective validation.

### 2.7. Gene Set Enrichment Analysis for Biomarker-Associated Pathways

To explore pathways associated with the candidate biomarkers, GSEA was performed using the clusterProfiler R package (version 4.7.1.001) with a significance threshold of adj.*p* < 0.05. Correlations between each biomarker and all other genes in GSE89408 were evaluated, and genes were prioritized according to their correlation coefficients. The reference gene set c2.cp.kegg.v2024.1.Hs.symbols.gmt was obtained from the MSigDB database (https://www.gsea-msigdb.org/gsea/msigdb, accessed on 26 November 2024).

### 2.8. Creation of Regulatory Networks and Drug Prediction

To further investigate predicted interaction networks related to the candidate biomarkers, miRNAs predicted to target candidate biomarkers, lncRNAs predicted to be associated with these miRNAs, and putative transcription factors (TFs) predicted to regulate candidate biomarkers were collected using the miRNet database (https://www.mirnet.ca/miRNet/home.xhtml, accessed on 28 May 2025), the Starbase database (https://rnasysu.com/encori/, accessed on 28 May 2025) (threshold: clipExpNum > 60), and the JASPAR database (https://jaspar.elixir.no/, accessed on 28 May 2025). Moreover, DGIdb was used to explore predicted drug–gene associations related to the candidate biomarkers. All prediction findings were imported into Cytoscape software (version 3.7.1) to visualize the lncRNA–miRNA–mRNA network, the TF–mRNA network, and the drug–mRNA network [16].

### 2.9. Prioritization of the Lead Fibroblast-Associated Marker

To delineate biomarker expression among cell types, all cells were stratified into high- and low-SASP score groups based on the average SASP score. We subsequently assessed, for each cell type, whether biomarker expression differed significantly between these two groups. Moreover, associations between biomarkers and SASP score were further assessed. The gene with high expression in fibroblasts, significant differences between high- and low-score groups, and a stronger correlation with SASP score was prioritized as the lead biomarker for downstream exploratory analyses. According to the expression of the lead biomarker, fibroblasts were categorized into the lead biomarker-high fibroblast group and the lead biomarker-low fibroblast group. A differential expression analysis was then performed between these two subgroups, followed by GSEA on the identified DEGs to investigate associated functional pathways (|NES| > 1, *p* < 0.05).

### 2.10. Cellular Communication Analysis

To investigate cell–cell communication patterns involving fibroblasts when they highly express the lead biomarker and when they express it at lower levels, cellular communication analysis was performed by the computeCommunProb function in CellChat (version 1.6.1) with default parameters. Moreover, potential ligand–receptor interactions across all cells were evaluated, with particular focus on interactions between the corresponding fibroblast subpopulations and other cell subpopulations. Significant ligand–receptor interactions were defined as those with a *p*-value < 0.05, calculated based on permutation tests (*n* = 100 permutations). With lead biomarker-high and lead biomarker-low fibroblast groups as sender cells, differential ligand–receptor interactions were identified (threshold settings: |log_2_FC| > 1 and *p*-value < 0.05). The CellChat reference framework was used for this analysis [17].

### 2.11. Pseudotime Analysis

To investigate the expression of the lead biomarker during fibroblast differentiation-related states, pseudotime analysis of fibroblasts was performed using the monocle R package (version 2.26.0) [18]. Additionally, the CytoTRACE algorithm [19] (version 0.3.3) was employed to create a trajectory illustrating the progression from low- to high-differentiation levels.

### 2.12. RT-qPCR Assessment

Human rheumatoid arthritis synovial fibroblasts (HRaSF-SV40, SNL-596; SUNNCELL, Wuhan, China) and human synovial cells (HS, CP-H094; Procell, Wuhan, China) were purchased from the indicated suppliers. These cell types were maintained in specialized culture media and cultured in an atmosphere with 5% CO_2_ at 37 °C. HS and HRaSF-SV40 cells were cultured in parallel and underwent the same batch of treatment, RNA extraction, reverse transcription, and RT-qPCR processing. Total RNA was extracted from cell samples using FastPure Complex Tissue/Cell Total RNA Isolation Kit (Vazyme, RC113-01, Nanjing, China), and its concentration was quantified using a Nanodrop-500 (Allsheng, nano-500, Hangzhou, China). RNA was then reverse transcribed into cDNA with ABScript III RT Master Mix for RT-qPCR with gDNA Remover (Abclonal, RK20429, Wuhan, China). RT-qPCR was conducted using Genious 2X SYBR Green Fast RT-qPCR Mix (Abclonal, RK21205, Wuhan, China) on an Archimed R4 real-time PCR system. The expression of biomarkers was normalized with *GAPDH* and analyzed by the 2^−ΔΔCt^ method. GAPDH was used only as a normalization reference for RT-qPCR because it showed stable Ct values across the cell-culture samples used in the validation experiment; however, it was not retained as a candidate biomarker because it lacked external reproducibility and has a housekeeping function. For each target gene in each group, three technical replicate wells were analyzed within each biological replicate. For biological reproducibility, three independent biological replicates were performed using cells from different passage numbers (passages 2, 4, and 6). For each biological replicate, HRaSF-SV40 and HS cells were thawed from separate frozen vials, cultured independently, and processed on different days. The primer sequences are listed in Table 1.

### 2.13. Statistical Analysis

All statistical analyses were conducted in R (version 4.2.2). Spearman correlation analysis was used to identify SASP-positive-related genes. Differential expression analysis in GSE89408 was performed using DESeq2, and adjusted *p*-values were used to control for multiple testing. Between-group expression comparisons in transcriptomic datasets were assessed using the Wilcoxon rank-sum test unless otherwise specified. Logistic regression models were evaluated in GSE89408 by 5-fold stratified cross-validation and externally validated in GSE77298 using receiver operating characteristic (ROC) analysis and area under the curve (AUC). External 95% confidence intervals for AUC were estimated using bootstrap resampling stratified by RA/control status. Enrichment analyses were evaluated using adjusted *p*-values, with adjusted *p* < 0.05 considered statistically significant. For other computational and cohort-level analyses, *p* < 0.05 was considered statistically significant. RT-qPCR data were summarized as mean ± SD from three independent biological replicates, each measured in technical triplicate wells, to provide supportive cell-culture-level evidence.

## 3. Results

### 3.1. Fibroblasts Showed Relatively High SASP-Related Scores Within the RA Single-Cell Dataset

**Figure 1:**
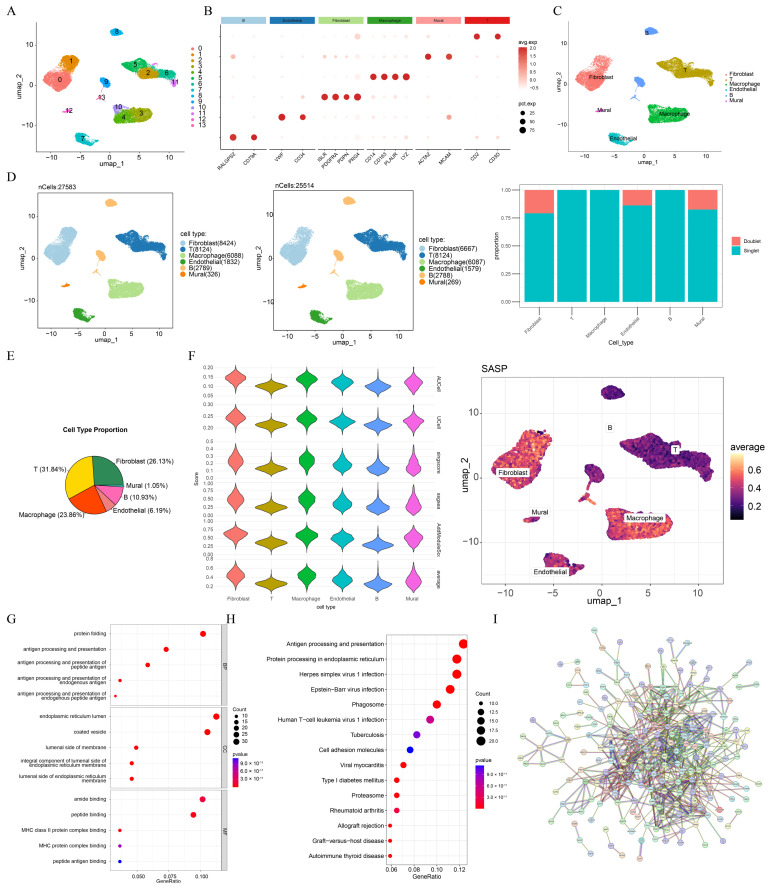
Fibroblasts showed relatively high SASP-related transcriptional scores within the RA single-cell dataset. (**A**) UMAP diagram showing the distribution of the 14 independent clusters. (**B**) Heatmap showing the expression of markers in cell types. (**C**) UMAP diagram showing the distribution of the 6 cell types. (**D**) Exclusion of doublets. A total of 2069 high-confidence doublets (7.5% of the initial 27,583 cells/nuclei) were removed, leaving 25,514 cells for downstream analysis. From left to right: UMAP diagram showing the distribution of the 6 cell types before removing doublets; UMAP diagram showing the distribution of the 6 cell types after removing doublets; the bar chart showing the proportion of doublets in each cell type: fibroblasts, 20.86%; macrophages, 0.02%; endothelial cells, 13.81%; B cells, 0.04%; and mural cells, 17.48%. (**E**) The proportion of each cell type. (**F**) The senescence-associated secretory phenotype (SASP) score of each cell type within the RA single-cell dataset. From left to right: violin chart; UMAP diagram. (**G**,**H**) Enrichment analysis for up-regulated genes in fibroblasts between high- and low-score groups. (**G**) Gene Ontology (GO) analysis results. (**H**) Kyoto Encyclopedia of Genes and Genomes (KEGG) analysis results. (**I**) The protein–protein interaction (PPI) network.

After the QC, a total of 27,583 cells and 36,601 genes remained for further analysis to guarantee the integrity and dependability of the single-cell analysis. Through an unsupervised cluster analysis, 14 clusters were identified, which were annotated to six major cell types: B cells, T cells, and macrophages (immune cells), as well as endothelial cells, mural cells, and fibroblasts (stromal cells) (Figure 1A–C). After removing the doublets, a total of 2069 (7.5%) high-confidence doublets were excluded, leaving 25,514 cells for the subsequent analysis (Figure 1D). The doublet removal rates varied across the cell types: fibroblasts, 20.86%; macrophages, 0.02%; endothelial cells, 13.81%; B cells, 0.04%; and mural cells, 17.48%. Among these cell types, the proportion of T cells was the highest (31.84%), followed by fibroblasts (26.13%) (Figure 1E). Through evaluating the SASP score, we found that, within this RA single-cell dataset, fibroblasts showed relatively higher SASP-related scores than the other major cell populations (Figure 1F). Consistent results were obtained in the validation cohort (Figure S1). Therefore, fibroblasts were selected for downstream exploratory analysis. Between the high- and low-score groups, 292 up-regulated genes were discovered, and these genes were significantly enriched in 1166 GO items (906 GO BP items, 141 GO CC items, and 119 GO MF items) and 73 KEGG pathways (Table S1), including protein folding (GO-BP), endoplasmic reticulum lumen (GO-CC), MHC class II protein complex binding (GO-MF), and antigen processing and presentation (KEGG) (Figure 1G,H). The PPI network included 202 nodes and 643 edges, among which B2M had interactions with 21 proteins, including CD74, CANX, HLA-F, and others (Figure 1I).

### 3.2. Cross-Cohort Evaluation Identified a Reproducible SASP-Related Three-Gene Candidate Panel

**Figure 2:**
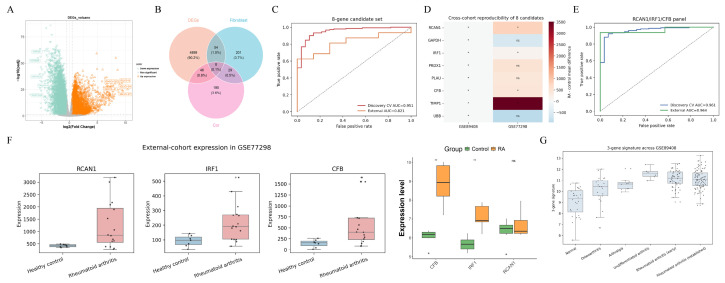
Identification and cross-cohort evaluation of SASP-related biomarkers. (**A**) Volcano plot showing the differentially expressed genes (DEGs) between RA and normal samples in GSE89408. (**B**) Venn diagram showing the intersections among DEGs between RA and control groups, SASP-positive-related genes, and up-regulated genes between high- and low-score groups for fibroblasts. (**C**) ROC curves for the 8-gene candidate set in discovery cross-validation and external evaluation. (**D**) Cross-cohort reproducibility heatmap for the initial 8-gene candidate set. (**E**) ROC curves for the *RCAN1/IRF1/CFB* three-gene candidate panel in discovery cross-validation and external evaluation. (**F**) External-cohort expression of *RCAN1*, *IRF1*, and *CFB* in GSE77298 and GSE7307. (**G**) Distribution of the *RCAN1/IRF1/CFB* three-gene signature across diagnostic categories in GSE89408. ns: not significant, * *p* < 0.05, ** *p* < 0.01, *** *p* < 0.001, **** *p* < 0.0001.

The correlation analysis between the average SASP score and the SASPRGs identified 278 SASP-positive-related genes (Table S2). The differential expression analysis in GSE89408 comparing the RA and normal samples uncovered 5007 genes with elevated expression (Figure 2A). Through intersections among the DEGs between the RA and control groups, the SASP-positive-related genes, and the up-regulated genes between the high- and low-score groups for fibroblasts, eight candidate genes were obtained: *RCAN1*, *GAPDH*, *IRF1*, *PRDX1*, *PLAU*, *CFB*, *TIMP1*, and *UBB* (Figure 2B). The cross-cohort evaluation showed that the full eight-gene candidate set yielded a 5-fold cross-validated AUC of 0.951 in GSE89408 and an external AUC of 0.821 in GSE77298 (Figure 2C). The candidate-gene reproducibility analysis showed that *RCAN1*, *IRF1*, *CFB*, and *TIMP1* remained significantly elevated in RA in both the datasets, with the RA–control differences in the same direction across the cohorts, whereas *GAPDH*, *PRDX1*, *PLAU*, and *UBB* were not reproduced in the external cohort (Figure 2D). Given the housekeeping nature of *GAPDH* and its lack of external reproducibility, *GAPDH* was excluded from further prioritization. We next evaluated all 15 non-empty panels derived from the four reproducibly elevated genes (Table S3). Among these combinations, the RCAN1/IRF1/CFB panel achieved the highest 5-fold cross-validated AUC in GSE89408 (0.961) and tied for the highest external AUC in GSE77298 (0.964; 95% CI 0.866–1.000) (Figure 2E). Although *IRF1/TIMP1* also tied for the highest external AUC, its discovery cross-validated AUC was substantially lower than that of *RCAN1/IRF1/CFB*, and it was therefore not retained. The four-gene panel including *TIMP1* showed the same external AUC and confidence interval but a slightly lower cross-validated AUC, indicating no measurable external-performance gain from adding *TIMP1*. Therefore, although *TIMP1* was externally reproducible, the more parsimonious *RCAN1/IRF1/CFB* panel was retained for the subsequent analyses. Their expression patterns were further evaluated in the primary external evaluation cohort GSE77298 and the additional small supportive cohort GSE7307 (Figure 2F). In GSE7307, *IRF1* and *CFB* exhibited directionally consistent upregulation in the RA synovium relative to the healthy controls, concordant with the patterns observed in GSE89408 and GSE77298. In contrast, *RCAN1* showed the same overall direction but did not reach statistical significance in this cohort, likely because of the limited sample size (six RA vs. five controls). Therefore, GSE7307 provides supportive expression-level evidence, particularly for *IRF1* and *CFB*, but should not be interpreted as a definitive validation of the full three-gene panel. Across the broader disease spectrum of GSE89408, the three-gene signature remained significantly higher in early RA and established RA than in normal synovium (both *p* < 1 × 10^−2^), while also exceeding normal levels in osteoarthritis, arthralgia, and undifferentiated arthritis (Figure 2G). The cross-cohort candidate-gene reproducibility and the model-performance summaries are provided in Tables S3 and S4.

### 3.3. Biomarker-Associated Pathway Enrichment

**Figure 3:**
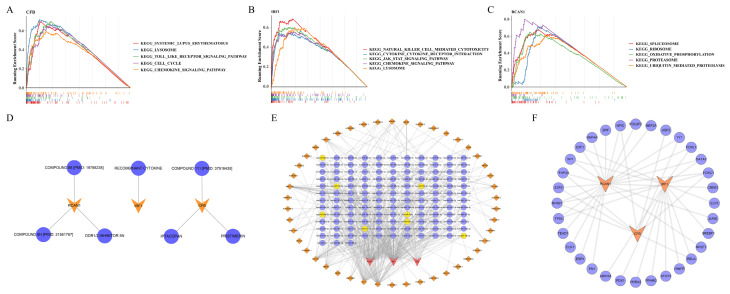
Gene set enrichment analysis for biomarker-associated pathways and predicted interaction networks. (**A**–**C**) Gene set enrichment analysis for *CFB*, *IRF1*, and *RCAN1*. (**D**) Predicted drug–gene association network. The orange nodes represent biomarkers and the blue nodes represent drugs. (**E**) Predicted lncRNA–miRNA–mRNA regulatory network. The red nodes represent biomarkers, the blue nodes represent miRNAs, the orange nodes represent lncRNAs, and the yellow nodes represent miRNAs regulating three candidate biomarkers. (**F**) Predicted transcription factor (TF)–mRNA regulatory network. The orange nodes represent biomarkers and the blue nodes represent TFs.

To explore the pathways associated with the candidate biomarkers, a GSEA was carried out. The results indicated that *CFB* was significantly enriched in 92 KEGG pathways, *IRF1* in 61 KEGG pathways, and *RCAN1* in 64 KEGG pathways (Table S5). Moreover, the top five KEGG pathways for each biomarker ranked by significance are displayed in Figure 3A–C. Through a comprehensive analysis, we found that these biomarkers were all significantly associated with 35 KEGG pathways, predominantly immune-related pathways, including the B cell receptor signaling pathway, the Toll-like receptor signaling pathway, and the T cell receptor signaling pathway, as well as cellular process-related pathways such as lysosome, apoptosis, and cell cycle, and genetic information processing-related pathways such as ubiquitin-mediated proteolysis, spliceosome, proteasome, and DNA replication. These findings indicate that the biomarkers were associated with a broad range of KEGG pathways.

### 3.4. Predicted Interaction Networks and Drug–Gene Associations of Biomarkers

Through the miRNet database, a total of 297 miRNAs predicted to target the candidate biomarkers were identified, and 135 lncRNAs predicted to be associated with these miRNAs were obtained from the Starbase database. Through the JASPAR database, a total of 32 putative TFs predicted to regulate the candidate biomarkers were identified. Through the DGIdb database, predicted drug–gene associations were identified for *RCAN1* (COMPOUND 66, DDR1/2 INHIBITOR 5N, and COMPOUND 8H), *IRF1* (recombinant cytokine), and *CFB* (COMPOUND 111, IPTACOPAN, and PRISTIMERIN). The drug–mRNA network included 10 nodes and 7 edges (Figure 3D). Through integration, the lncRNA–miRNA–mRNA regulatory network included 3 mRNAs, 135 miRNAs, 46 lncRNAs, and 445 edges (Figure 3E). The representative predicted relationships included NEAT1-hsa-let-7a-5p-*RCAN1*, SNHG1-hsa-miR-101-3p-*IRF1*, and MALAT1-hsa-miR-1271-5p-*CFB*. The TF–mRNA network included 35 nodes and 46 edges (Figure 3F). Among these TFs, PRRX2 and NFKB1 were connected with all three biomarkers in the predicted TF–mRNA network.

### 3.5. CFB Was Prioritized as the Lead Fibroblast-Associated SASP-Related Marker

**Figure 4:**
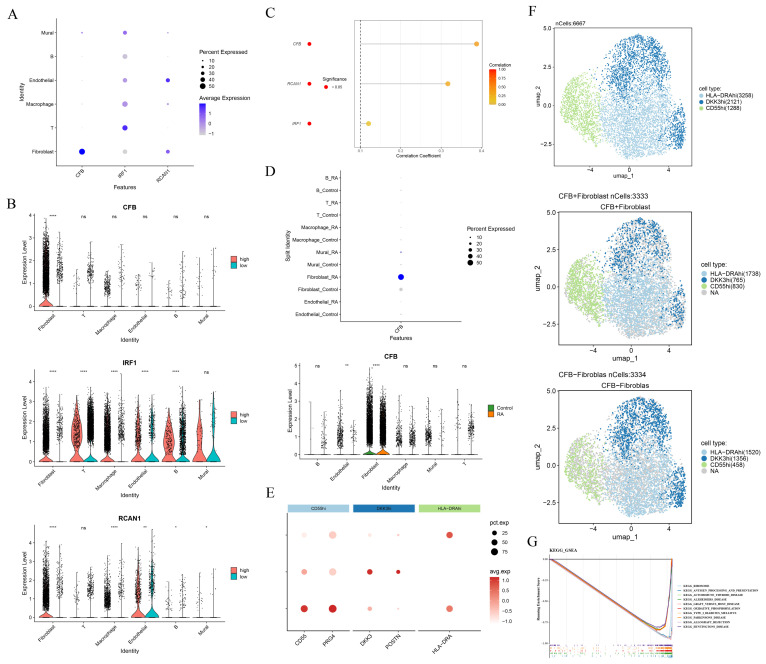
CFB was identified as the lead fibroblast-associated SASP-related marker. (**A**) The expression of biomarkers across cell types. (**B**) The expression of biomarkers between high- and low-score groups in each cell type. (**C**) Correlations between biomarkers and SASP score. (**D**) The expression of *CFB* in each cell type of RA and control samples. (**E**) The secondary clustering analysis of fibroblasts. (**F**) The proportion of cell subpopulations of fibroblasts in *CFB*-high and *CFB*-low fibroblasts. (**G**) GSEA for DEGs between *CFB*-high fibroblasts and *CFB*-low fibroblasts. ns: not significant, * *p* < 0.05, ** *p* < 0.01, *** *p* < 0.001, **** *p* < 0.0001.

Among the six cell types, the expression of *CFB* was the highest in fibroblasts, *IRF1* in T cells, and *RCAN1* in endothelial cells (Figure 4A). Across the high- and low-score groups (across all the cells), fibroblasts showed marked differences in the expression of all three biomarkers (Figure 4B). These findings were further corroborated in the RA samples from GSE200815 within the GPL24676 validation setting (Figure S2). Moreover, the correlation between CFB and the SASP score was the strongest (Figure 4C). Therefore, CFB was prioritized as the lead fibroblast-associated SASP-related marker. An exploratory comparison between GSE243917 RA fibroblasts and GSE216651 healthy fibroblasts showed a higher average *CFB* expression and a higher percentage of CFB-expressing fibroblasts in the RA group (Figure 4D), but this analysis should be interpreted as a supportive rather than a definitive disease-specific validation because of cross-study integration and tissue-source differences. According to the expression of *CFB* in fibroblasts, the fibroblasts were categorized into *CFB*-high fibroblasts and *CFB*-low fibroblasts. To determine whether these two subgroups represent distinct cell subtypes or functional states, we performed secondary clustering of all the fibroblasts. The results showed that a total of three cell subpopulations were annotated: HLA-DRAhi sublining fibroblasts (*HLA-DRA*), DKK3hi sublining fibroblasts (*DKK3* and *POSTN*), and CD55hi lining fibroblasts (*CD55* and *PRG4*) (Figure 4E). The *CFB*-high and *CFB*-low fibroblasts were evenly distributed across all the identified fibroblast subclusters, with no distinct or unique fibroblast cell type specifically corresponding to either group (Figure 4F). These findings support the interpretation of *CFB*-high and *CFB*-low as state-associated differences rather than fully discrete fibroblast identities.

The GSEA results demonstrated that the DEGs between the *CFB*-high and *CFB*-low fibroblasts were significantly enriched in 43 KEGG pathways, covering functional categories such as proteasome, innate immunity (NOD-like receptor signaling pathway), and immune effector function (natural killer cell-mediated cytotoxicity) (Figure 4G).

### 3.6. Pseudotime Trajectory Analysis of CFB-Expressing Fibroblasts

**Figure 5:**
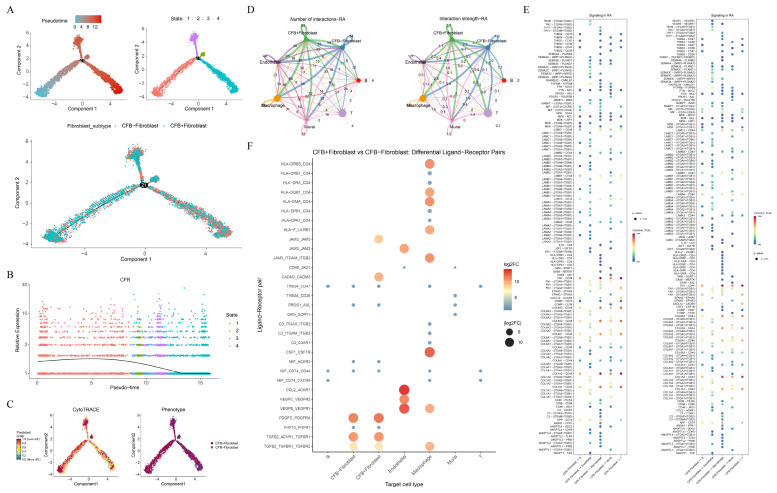
Pseudotime analysis and inferred cell–cell communication. (**A**) Pseudotime trajectory analysis of fibroblasts. From left to right: cells are colored according to pseudotime value, inferred state, and *CFB*-high/*CFB*-low grouping. (**B**) *CFB* expression in different stages. (**C**) CytoTRACE analysis showing CytoTRACE scores for *CFB*-high and *CFB*-low fibroblasts. (**D**) Inferred cell–cell communication network illustrating the number and strength of interactions among cells. (**E**) The ligand–receptor pairs between *CFB*-high fibroblasts and other cells, as well as *CFB*-low fibroblasts and other cells. (**F**) Comparison of the significant ligand–receptor pairs between *CFB*-high fibroblasts and *CFB*-low fibroblasts.

Subsequently, a pseudotime analysis of the fibroblasts was conducted to examine how *CFB* expression varied across the pseudotime-associated fibroblast states. The inferred trajectory displayed a branched architecture rather than a simple linear path, with an inferred root-associated region and two downstream branches (Figure 5A). *CFB* expression was higher near the inferred root-associated region and decreased across both branches (Figure 5B). The CytoTRACE analysis further indicated that the *CFB*-high fibroblasts showed features consistent with a less differentiated or more plastic state than the *CFB*-low fibroblasts (Figure 5C). Since a branched pseudotime topology does not by itself prove a single biological differentiation route, these findings are interpreted as exploratory evidence that *CFB* is associated with pseudotime-related fibroblast states rather than as definitive proof of a single continuous differentiation process.

### 3.7. CFB Expression Status Was Associated with Altered Inferred Fibroblast Cell–Cell Communication

Additionally, an examination of cellular communication demonstrated enhanced cell–cell interactions, both in quantity and intensity, between the *CFB*-high fibroblast group and other cells, compared with the *CFB*-low fibroblast group (Figure 5D). As shown in Figure 5E, we analyzed the ligand–receptor interactions between the *CFB*-high or *CFB*-low fibroblasts and other cell types, with these two fibroblast states serving as the sender cells. To systematically compare the communication characteristics of the two fibroblast subsets, a differential ligand–receptor interaction analysis was performed with these two cell states as the signal senders. A total of 51 ligand–receptor pairs showed significant differences in communication strength (Table S6) (Figure 5F). Specifically, the *CFB*-high fibroblasts exhibited markedly enhanced interactions with endothelial cells (e.g., CCL2-ACKR1, VEGFB-VEGFR1, VEGFC-VEGFR2) and macrophages (e.g., CSF1-CSF1R, HLA-DRB5-CD4, HLA-DMA-CD4). In contrast, ligand–receptor interactions such as THBS4-CD47 and PROS1-AXL showed stronger signaling in the CFB-low fibroblasts. Collectively, these findings suggest that the *CFB* expression status is associated with altered inferred cell–cell communication patterns in synovial fibroblasts, with the CFB-high fibroblasts showing a stronger predicted signaling toward endothelial and immune cells.

### 3.8. RT-qPCR Assessment Supported the Upregulation of IRF1, CFB, and RCAN1

**Figure 6:**
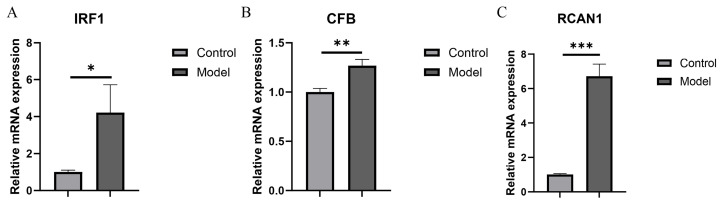
RT-qPCR assessment of candidate biomarkers. (**A**–**C**) Relative mRNA expression levels of *IRF1*, *CFB*, and *RCAN1* in human synovial cells and human rheumatoid arthritis synovial fibroblasts. Three independent biological replicates were performed using cells from different passage numbers (passages 2, 4, and 6). Data are presented as mean ± SD of three biological replicates, with each biological replicate measured in technical triplicates. * *p* < 0.05, ** *p* < 0.01, *** *p* < 0.001.

To further evaluate the computational findings, we conducted RT-qPCR using human synovial cells and human rheumatoid arthritis synovial fibroblasts from three independent biological replicates (cells from passages 2, 4, and 6). Consistent with the transcriptomic analyses, *IRF1*, *CFB*, and *RCAN1* all showed higher relative mRNA expression levels in the rheumatoid arthritis-related cell samples than in the synovial cell group (Figure 6, Table S7). These RT-qPCR results provide preliminary mRNA-level support for the increased expression of the three biomarkers.

## 4. Discussion

Based on single-cell analysis, multi-category bulk transcriptomics, external evaluation, and RT-qPCR support, this study supports the involvement of fibroblasts as an SASP-associated cell population in rheumatoid arthritis. Within the RA single-cell dataset, fibroblasts showed relatively high SASP-related scores compared with the other major cell populations. The cross-cohort results further indicate that a compact *RCAN1/IRF1/CFB* panel provides more stable discrimination than the initial broader candidate set in the current data context.

Synovial fibroblasts are pivotal in RA progression due to their activated phenotypes, mainly manifested as proinflammatory factor secretion and joint matrix degradation. Prior studies have demonstrated that fibroblast-derived SASP factors, such as IL-6 and MMPs, play pivotal roles in RA pathogenesis [4,20]. In our data, *CFB* showed the strongest association with fibroblasts and the SASP score, while the pathway enrichment analysis linked *CFB*-associated genes to immune and inflammatory programs. Given previous evidence that *CFB* is an essential regulator of the alternative complement pathway [21,22], these findings suggest that *CFB* may participate in inflammatory amplification within the RA synovium. Likewise, the enrichment of the pathways related to neutrophil extracellular trap formation and Fc gamma receptor-mediated phagocytosis should be interpreted as hypothesis-generating observations that are broadly consistent with prior reports on inflammatory activation in RA [23]. In addition, the pseudotime analysis suggested that *CFB*-high fibroblasts may preferentially occupy earlier differentiation-related states, whereas previous single-cell studies have highlighted substantial fibroblast heterogeneity in the RA synovium [24].

The GSEA suggested that the identified biomarkers, *CFB*, *IRF1*, and *RCAN1*, were associated with pathways relevant to RA biology, including NF-κB signaling, cytokine–cytokine receptor interaction, and T cell activation-related pathways. NF-κB signaling is a well-established pathogenic pathway in RA and has been implicated in inflammatory cytokine production, synovial fibroblast activation, matrix-degrading enzyme expression, and joint destruction [25,26]. In addition, previous studies have linked METTL3-mediated regulation and JAK-STAT signaling to the inflammatory activation of fibroblast-like synoviocytes and cytokine-related RA pathobiology [27,28]. In the present study, these pathway associations should be interpreted as literature-supported biological interpretations of the enrichment results rather than experimentally demonstrated mechanisms. Although *CFB* showed a strong fibroblast-associated expression pattern and was linked to immune and inflammatory pathway enrichment, our data do not establish direct causal crosstalk among *CFB*, NF-κB, JAK-STAT, complement activation, STAT3 activation, glycolytic reprogramming, or METTL3. Therefore, the observed associations may help prioritize future mechanistic studies, but they should not be regarded as proof of a self-amplifying inflammatory loop or a validated therapeutic mechanism.

Several limitations should be acknowledged. First, we have integrated extra single-cell datasets to validate the key findings, but all the single-cell results were generated from public databases. These cellular characteristics still need to be confirmed using clinical samples from independent cohorts. Second, two external bulk cohorts were used for the external evaluation, but both had small sample sizes. In particular, GSE7307 was used only as an additional supportive expression–validation cohort, and the nonsignificant RCAN1 result in this cohort should be interpreted cautiously. Larger independent synovial cohorts are needed for the definitive validation of the three-gene signature. Third, because the candidate genes were preselected before the cross-validation, the discovery cross-validated AUC should be interpreted as an internal performance estimation rather than a fully unbiased nested validation. Fourth, the RT-qPCR assessment provided supportive mRNA-level evidence, whereas protein-level and functional experiments remain necessary. Finally, the predicted regulatory and drug–gene networks require experimental validation.

## 5. Conclusions

This study highlighted fibroblasts as a cell population showing relatively high SASP-related transcriptional scores within the RA single-cell dataset and identified *RCAN1*, *IRF1*, *CFB*, and *TIMP1* as reproducibly elevated SASP-related candidate genes across the evaluated bulk cohorts. Among all the panels derived from these four reproducibly elevated genes, *RCAN1/IRF1/CFB* achieved the highest cross-validated AUC and tied for the highest external AUC, whereas adding *TIMP1* did not improve the external AUC or confidence interval. *RCAN1*, *IRF1*, and *CFB* were therefore retained as the final three-gene candidate panel. Among them, *CFB* showed the strongest fibroblast-associated expression pattern in our analyses. These observations provide cross-cohort computational evidence relevant to rheumatoid arthritis biology, while further protein-level, functional, and controlled single-cell validation studies remain necessary.

## Figures and Tables

**Table 1:** Primer sequences used for RT-qPCR.

Gene	Forward Primer (5′–3′)	Reverse Primer (5′–3′)
*GAPDH*	CTGGGCTACACTGAGCACC	AAGTGGTCGTTGAGGGCAATG
*CFB*	CAGGAAGGTGGCTCTTGGAG	CCCATCCTCAGCATCGACTC
*IRF1*	CCTTAAGAACCCGGCAACCT	TGCATCTCTAGCCAGGGTCT
*RCAN1*	GATGGAGGACGGCGTGG	TCAATGAAGCTCCAGTCGCC

## Data Availability

The public datasets analyzed in this study are available from the Gene Expression Omnibus under accession numbers GSE89408, GSE77298, GSE7307, GSE243917, GSE200815, and GSE216651. The cross-cohort candidate-gene reproducibility and all panel-combination results are provided in Tables S3 and S4. RT-qPCR Cq values and relative-expression calculations are provided in Table S7.

## Supplementary Materials

The following supporting information can be downloaded at: https://www.mdpi.com/article/10.3390/genes17070736/s1, Table S1: The list of Gene Ontology (GO) items and Kyoto Encyclopedia of Genes and Genomes (KEGG) pathways enriched for up-regulated genes between high- and low-score groups in fibroblasts; Table S2: The list of SASP-positive-related genes; Table S3: Performance of all possible panels derived from the four reproducible genes (*RCAN1*, *IRF1*, *CFB*, and *TIMP1*); Table S4: Cross-cohort candidate-gene reproducibility summary; Table S5: The list of KEGG pathways enriched for biomarkers through GSEA; Table S6: The list of ligand-receptor with significant differences between CFB-high fibroblasts and CFB-low fibroblasts; Table S7: RT-qPCR Cq values and relative-expression calculations; Figure S1: The single-cell analysis in the validation cohort (GSE200815 and GSE216651); Figure S2: The validation of biomarker expression in GSE200815.

## Abbreviations

The following abbreviations are used in this manuscript:

RA	rheumatoid arthritis
SASP	senescence-associated secretory phenotype
DMARDs	disease-modifying antirheumatic drugs
IPFP	infrapatellar fat pad
PCA	principal component analysis
FC	fold change
GO	Gene Ontology
KEGG	Kyoto Encyclopedia of Genes and Genomes
PPI	protein–protein interaction
BH	Benjamini–Hochberg
FDR	false discovery rate
TFs	transcription factors
ROC	receiver operating characteristic
AUC	area under the curve
