# Supplementary material for: Integrated Bulk and Single-Cell Transcriptomic Analysis Identifies a Reproducible SASP-Related Three-Gene Panel and Prioritizes CFB as a Fibroblast-Associated Marker in Rheumatoid Arthritis
Source: Genes (Basel). 2026 Jun 26;17(7):736. doi: 10.3390/genes17070736 (PMC13409691; doi:10.3390/genes17070736)
Supplement: Supplementary file 1 [file genes-17-00736-s001.zip › Supplementary Figures.pdf]

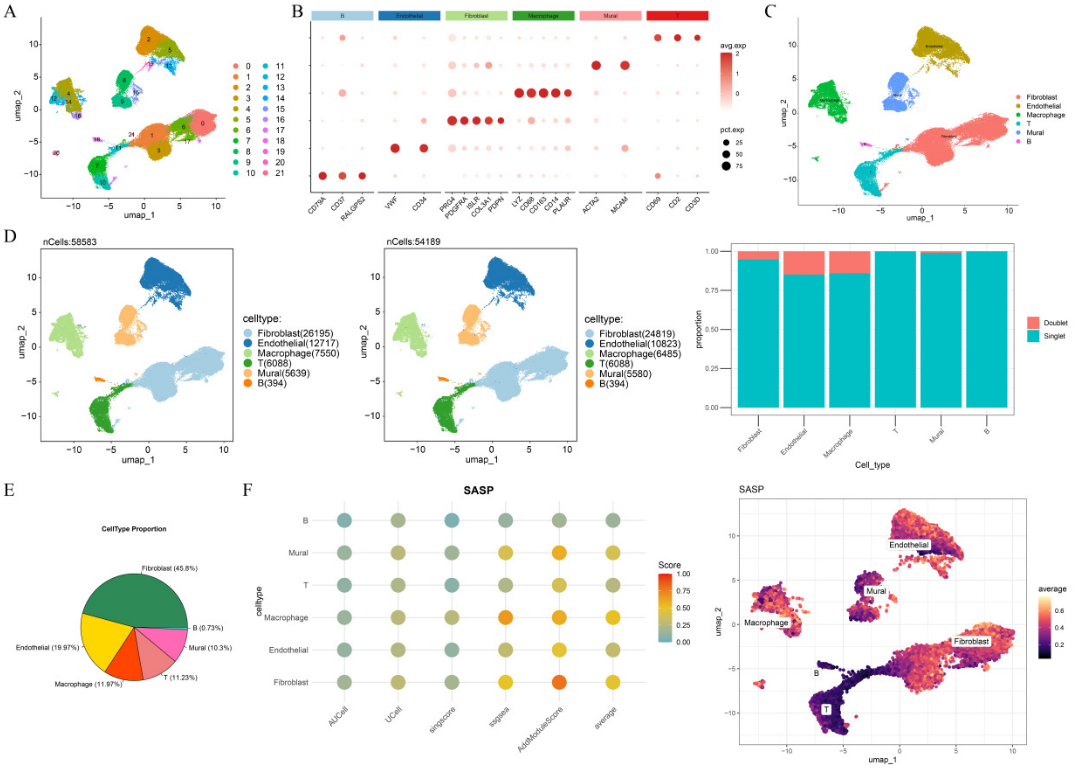

Supplementary Figure S1 The single-cell analysis in validation cohort (GSE200815 and GSE216651). (A) UMAP diagram showing the distribution of the 21 independent clusters. (B) Heatmap showing the expression of markers in cell types. (C) UMAP diagram showing the distribution of the 6 cell types. (D) Exclusion of doublets. From left to right: UMAP diagram showing the distribution of 6 cell types before removing doublets; UMAP diagram showing the distribution of 6 cell types after removing doublets; the bar chart showing the proportion of doublets in each cell type. (E) The proportion of each cell type. (F) The senescence-associated secretory phenotype (SASP) score of each cell type within the integrated validation cohort based on GSE200815 and GSE216651. From left to right: the violin chart; the UMAP diagram.

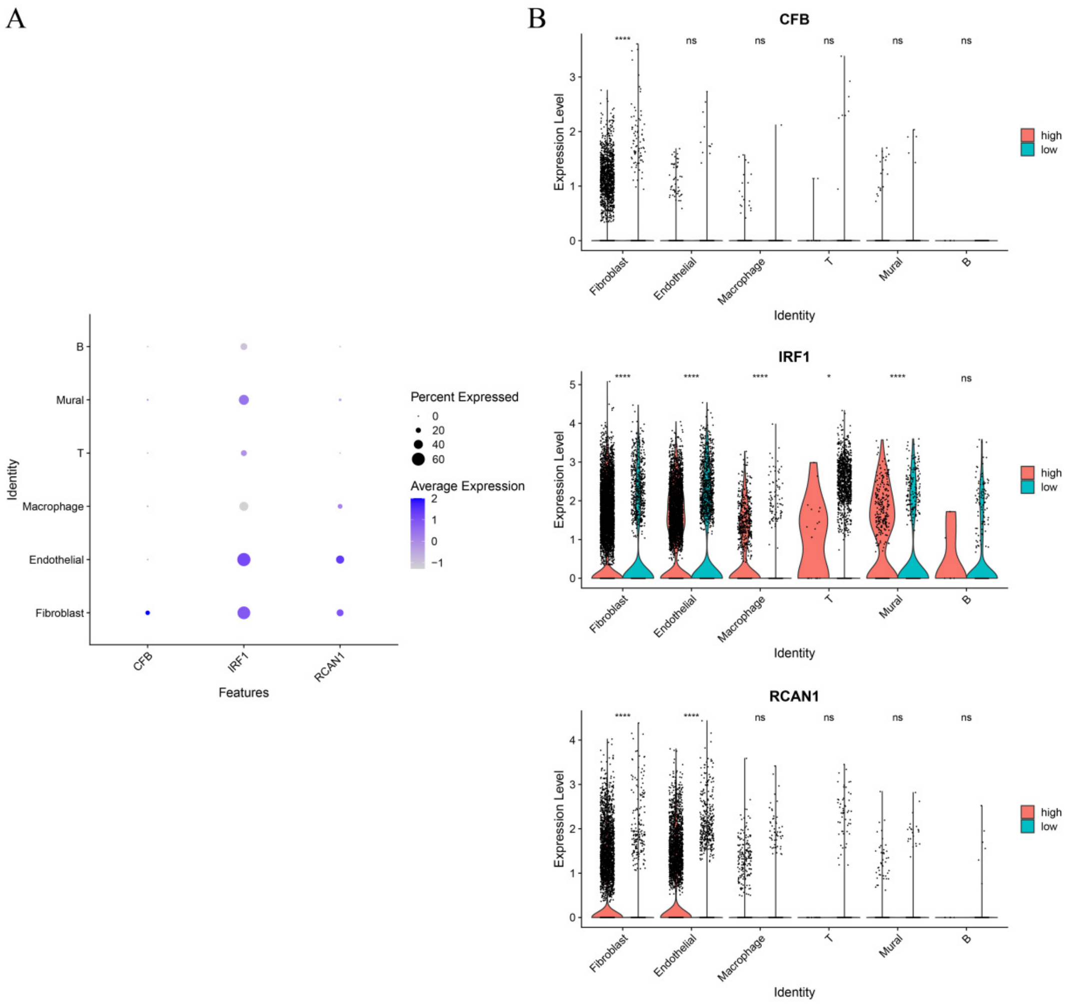

Supplementary Figure S2 The validation of biomarker expression in GSE200815. (A) The expression of biomarkers across cell types. (B) The expression of biomarkers between high- and low-SASP score groups in each cell type.
